# Elevated homocysteine is negatively correlated with plasma cystathionine β‐synthase activity in givosiran‐treated patients

**DOI:** 10.1002/jmd2.12416

**Published:** 2024-04-29

**Authors:** Mark A. Keibler, Gautham V. Sridharan, Marianne T. Sweetser, Simina Ticau

**Affiliations:** ^1^ Research Alnylam Pharmaceuticals Cambridge Massachusetts USA; ^2^ Clinical Research Alnylam Pharmaceuticals Cambridge Massachusetts USA

**Keywords:** Acute hepatic porphyria (AHP), cystathionine β‐synthase (CBS), givosiran, homocysteine, hyperhomocysteinemia, liquid chromatography‐mass spectrometry (LC–MS), noninvasive diagnostics

## Abstract

Givosiran is a subcutaneously administered, liver‐targeted RNA interference (RNAi) therapeutic that has been approved for treating acute hepatic porphyria (AHP). Elevation in plasma homocysteine (hyperhomocysteinemia) has been reported in AHP patients, and treatment with givosiran has been reported to further increase homocysteine levels in some patients. The mechanism of homocysteine elevation during givosiran treatment is unknown, but has been hypothesized to be mediated by a reduction in activity of cystathionine β‐synthase (CBS), which uses homocysteine as a substrate. A liquid chromatography‐tandem mass spectrometry‐based assay was adapted to measure circulating CBS activity. Using plasma collected from the Phase III ENVISION study, CBS activity was measured to directly evaluate whether it is associated with elevated homocysteine levels in givosiran‐treated patients. CBS activity was reduced following givosiran treatment and both homocysteine and methionine levels were inversely correlated with CBS activity. Following administration of a supplement containing vitamin B_6_, a cofactor for CBS, in four patients during the trial, plasma CBS activity was found to increase, mirroring a corresponding decrease in homocysteine levels. These results support the hypothesis that elevated homocysteine levels following givosiran treatment result from a reduction of CBS activity and that vitamin B_6_ supplementation lowers homocysteine levels by increasing CBS activity.

**Synopsis**

Plasma cystathionine β‐synthase activity is reduced in acute hepatic porphyria patients following givosiran treatment and is negatively correlated with homocysteine levels.

## 1 INTRODUCTION

Givosiran is a subcutaneously administered, liver‐targeted RNA interference (RNAi) therapeutic that has been approved for treating acute hepatic porphyria (AHP). AHP is comprised of four types of porphyria: acute intermittent porphyria (the most common, OMIM 176000), variegate porphyria (OMIM 176200), hereditary coproporphyria (OMIM 121300), and 5‐aminolevulinic acid (5‐ALA) hydratase deficiency (OMIM 612740).^1^, ^2^ AHP is a family of diseases caused by rare hereditary mutations in genes encoding for enzymes in heme biosynthesis.^3^, ^4^ In patients with AHP, neurotoxic intermediates in heme biosynthesis, 5‐ALA, and porphobilinogen (PBG), accumulate and induce acute neurovisceral attacks. Givosiran targets 5′‐aminolevulinic acid synthase 1 (5‐ALAS1; EC 2.3.1.37), the first and rate‐limiting step of heme biosynthesis.^3^, ^4^, ^5^ Clinical studies have demonstrated that givosiran treatment leads to sustained lowering of urinary ALAS1 mRNA, 5‐ALA, and PBG levels, and, in patients experiencing recurrent attacks, reduces the annualized attack rate compared with placebo.^6^, ^7^, ^8^

Elevation in plasma homocysteine (hyperhomocysteinemia) has been reported in AHP patients, and treatment with givosiran has been reported to further increase homocysteine levels in some patients.^9^, ^10^ Data from ENVISION, a Phase III clinical trial evaluating givosiran in AHP patients with ongoing attacks, showed an increase in plasma homocysteine following givosiran treatment at the population level but no correlation between plasma homocysteine levels and efficacy or safety of givosiran.^11^ In the general population, elevated homocysteine may only be a moderate predictor of cardiovascular, cerebrovascular, and thromboembolic disease.^12^, ^13^, ^14^ However, the clinical implications of homocysteine elevation in givosiran‐treated AHP patients are unclear.

Homocysteine can be converted to methionine via the remethylation pathway or to cystathionine via the transsulfuration pathway.^15^ Classical homocysteinuria (OMIM 236200) patients possess inactivating mutations in the gene encoding cystathionine β‐synthase (CBS; EC 4.2.1.22), the first enzyme in the transsulfuration pathway.^16^ Mutations in genes encoding other enzymes in the pathways can also contribute to hyperhomocysteinemia, as can deficiencies in vitamins (B_6_, B_12_, folate) that serve as cofactors for associated enzymes; supplementing these vitamins can help lower homocysteine levels.^17^

The mechanism of increased homocysteine levels following givosiran treatment is unclear, but current evidence suggests that it may be mediated by reduction in CBS activity. In support of this hypothesis, methionine, upstream of CBS and connected to homocysteine via the remethylation pathway, also increases in givosiran patients.^11^ Decreased activity of ALAS1, the first and rate limiting step in heme biosynthesis, may reduce levels of hepatic heme.^18^ CBS uses heme as a cofactor, although the exact role of heme in CBS function is not clear.^19^ Additionally, previous reports describe reductions in plasma homocysteine levels in givosiran patients following supplementation with vitamin B_6_, another cofactor for CBS.^11^, ^20^ Further, vitamin B_6_ supplementation has been long known to reduce homocysteine levels in a substantial portion of CBS‐deficient classical homocysteinuria patients,^16^, ^21^ potentially through a chaperoning role from pyridoxal 5′‐phosphate (PLP).^22^, ^23^

Measuring plasma CBS activity using a liquid chromatography‐tandem mass spectrometry (LC–MS/MS)‐based assay has been proposed as a diagnostic tool for detecting CBS deficiency.^24^, ^25^ Although CBS is intracellular, some leakage of the enzyme into circulation occurs, enabling its activity to be measured non‐invasively. This plasma‐based assay has shown significantly different CBS activity levels between healthy controls and CBS‐deficient patients, particularly those unresponsive to vitamin B_6_ supplementation, whose CBS activity was apparently undetectable using the assay.

Using plasma collected from the ENVISION study for exploratory biomarker analysis, we measured CBS activity to directly evaluate whether it is associated with elevated homocysteine in givosiran patients.

## 2 MATERIALS AND METHODS

### 2.1 ENVISION study design and sample collection

Plasma samples from AHP patients enrolled in the Phase III ENVISION study who consented to exploratory biomarker assessment and had baseline samples were analyzed for CBS activity.^6^ The study consisted of a 6‐month double‐blind period, in which patients were given either placebo or 2.5 mg/kg givosiran monthly, followed by an open‐label extension period, during which all patients were given givosiran. Exploratory biomarker samples were available for 41 of the total 46 patients assigned to the placebo arm and 45 of the total 48 patients assigned to the givosiran arm. Samples from each patient at baseline (study initiation), Month 6 (end of double‐blind period), and Month 12 (6 months into open‐label extension) were analyzed for CBS activity.

Additional plasma samples from four patients who were given a daily multivitamin supplement containing vitamin B_6_ (NORMOCIS 400, containing 400 μg folate [as 5‐methyl‐tetrahydrofolic acid], 3 mg vitamin B_6_ [as pyridoxine], 5 μg vitamin B_12_ [as cyanocobalamin], 2.4 mg vitamin B_2_ [as riboflavin], 250 mg betaine, and 12.5 mg zinc; IrisFarma) 32–34 months into the ENVISION study were also analyzed for CBS activity. These samples were collected at Months 24 and 36.

To prepare the plasma samples, whole blood was added to K_2_EDTA tubes and mixed immediately by gently inverting the tubes at least 8–10 times. Samples were centrifuged at 1500×g to 2000×g for 15 min until cells and plasma were separated. The plasma was then transferred into a labeled 2.0 mL cryovial and immediately frozen at −70°C or below. They were shipped frozen and stored at −70°C. Samples were stored roughly 7–50 months prior to analysis.

Method development and validation were performed using a single lot of commercially available human plasma with K_2_EDTA as an anticoagulant from BioIVT.

### 2.2 Homocysteine and methionine measurements

Homocysteine and methionine assessments utilized archival serum or plasma samples, respectively. Homocysteine levels were measured by Medpace Reference Labs using the Diazyme's Dual Reagent Enzymatic Homocysteine Assay on a Beckman Coulter chemistry analyzer. This assay had an upper quantification limit of 400 μM. The measured homocysteine concentration exceeded this limit for five samples, so these corresponding data points were excluded for the assessment of correlation between plasma CBS activity and homocysteine levels. Methionine levels were measured using an LC–MS/MS assay at ARUP Laboratories.

### 2.3 Chemicals

All chemicals were purchased from Sigma unless otherwise mentioned. D_3_‐L‐serine and D_4_‐DL‐cystathionine were purchased from Cambridge Isotope Laboratories. Sodium hydroxide (NaOH), hydrochloric acid (HCl), and formic acid were purchased from Fisher Scientific. Tris–HCl, pH 8.6 was purchased from Alfa Aesar.

### 2.4 CBS activity assay sample preparation

The conditions used for the plasma CBS assay were based on a protocol described by Krijt et al., as detailed below.

All steps were performed on ice or at 4°C unless otherwise mentioned. Plasma samples that had been stored at −80°C were thawed at 37°C for roughly 10 min, at which point they were gently vortexed and centrifuged at 2000×g for 5 min. All reaction components were frozen as aliquots at −80°C, aside from NaOH, and all cystathionine standards were frozen as aliquots at −20°C; these aliquots were also thawed at 37°C for roughly 10 min prior to the assay. All components of the CBS reaction mixture are given on a per‐sample basis, but in practice, bulk mixes of the initiation and partial reaction mixture (i.e., per‐sample volumes scaled by the total number of samples) were prepared prior to their addition to microwell plates.

An initiation mixture, containing 1.25 μL of 4.9 NaOH and 1.25 μL of 1180 mM homocysteine thiolactone, was pipetted into a microcentrifuge tube and vortexed. This mixture was then incubated at 37°C for 5 min to liberate free homocysteine. Next, 1.67 μL of a solution containing 3 M HCl and 300 mM Tris–HCl (pH 8.6) was added to neutralize the initiation mixture, followed by 0.83 μL of 60 mM dithiothreitol to maintain homocysteine in a reduced state.

A partial reaction mixture was prepared by pipetting 20 μL of a stock of several components (200 mM Tris–HCl [pH 8.6], 40 mM D_3_‐L‐serine, and 1 mM pyridoxal phosphate) to 5 μL of 5 mM S‐adenosylmethionine (SAM). As well, a quenching mixture was prepared by adding 200 μL per sample of methanol and 0.2 μL per sample of 500 μM D_4_‐cystathionine internal standard.

To 96‐microwell plate wells, 20 μL of each plasma sample (or cystathionine standard) was added, followed by 25 μL of the partial reaction mixture and then 5 μL of the initiation mixture. Next, the plates were briefly shaken on a plate shaker before being placed in an incubator set at 37°C. After 4 h, the plates were removed and placed on ice, and 200 μL of the ice‐chilled quenching mixture was added to each well to stop the reaction and precipitate proteins. The precise duration of the reaction was measured to the minute with a stopwatch and used to calculate reaction rates. These post‐quench plates were then mixed briefly on a plate shaker and then placed at −80°C overnight to promote further protein precipitation.

The following day, the plates were removed from the −80°C freezer, briefly mixed on a plate shaker, and then centrifuged at 4200×g for 5 min. Afterward, 200 μL supernatant was transferred to AcroPrep Advance 96‐well filter plates (Pall Corporation), and these plates were again centrifuged at 4200×g for 5 min with a 96‐well collection plate underneath. Finally, 50 μL of cleared filtrate was added to 150 μL of ultrapure water in new 96‐well plates, and this diluted filtrate was used for LC–MS/MS analysis.

### 2.5 LC–MS/MS analysis

Sample plates were analyzed using an ExionLC AD high‐performance liquid chromatography system (Sciex) coupled to a QTRAP 7500 mass spectrometer (Sciex). Cystathionine was chromatographically resolved by injecting 0.1–0.5 μL onto a SunFire C8 column (4.6 mm i.d. × 100 mm, 3.5 μm; Waters) with a VanGuard Cartridge Holder with a SunFire C8 VanGuard Cartridge (3.9 mm i.d. × 5 mm, 3.5 μm; Waters). An isocratic mobile phase of 60% water with 0.1% formic acid and 40% methanol with 0.1% formic acid was used. The mobile phase flow rate was set to 0.35 mL/min, and each sample acquisition lasted for 5 min.

Analytes were measured using positive ion mode. The multiple reaction monitoring (MRM) transitions 222.9 ➔ 134.0, 224.9 ➔ 134.0, and 226.9 ➔ 138.1 were used to detect monoisotopic, D_2_‐labeled, and D_4_‐labeled cystathionine, respectively, with collision energies of 20 V and both entrance and collision exit potentials of 10 V for each. The ion source gas 1 was set to 65 psi, the ion source gas 2 was set to 90 psi, the curtain gas was set to 40 psi, the source temperature was set to 575°C, the collision gas pressure was set to 8 psi, and the ion spray voltage was set to 1800 V.

### 2.6 Data analysis

LC–MS/MS data were acquired and processed using Sciex OS (Sciex). Peaks at 2.50 min were integrated for cystathionine quantification. After manual inspection of the integrated peaks, the peak areas were exported as a .csv file. This file was imported to Microsoft Office Excel 2016 (Microsoft Corporation). The ratio of the peak areas of the MRM transitions 222.9 ➔ 134.0 or 224.9 ➔ 134.0 and 226.9 ➔ 138.1 (i.e., monoisotopic or D_2_‐cystathionine to D_4_‐labeled cystathionine) to normalize abundances to internal standard. These ratios were then imported into GraphPad Prism 8.2.1, where the monoisotopic‐to‐D_4_‐cystathionine ratios from unlabeled cystathionine standards were used to construct a calibration curve. The calibration curve, which was weighted by the inverse of the standard concentrations, ranged from 100 to 0.01 μM across 2‐ and 10‐fold serial dilutions; its *R* ^2^ was at least 0.9990, covered the entire span of measured sample cystathionine ratios, and the measured concentrations of all external standards did not deviate from nominal concentrations by more than 15%. For plasma CBS activity assay samples, the D_2_‐to‐D_4_‐cystathionine ratios were fitted to the calibration curve to estimate D_2_‐cystathionine concentrations in the reaction mixture. These measured concentrations were then divided by the measured reaction duration and scaled by the 20 μL plasma analyzed to calculate CBS activity values in nmol L^−1^ h^−1^. (Interpolated concentrations in reaction mixtures quenched immediately after addition of the initiation mixture were found to range from 0.01 to 0.02 μM, so initial D_2_‐cystathionine concentrations were considered negligible in calculating CBS activity values.)

### 2.7 Statistical analysis

Multivariable linear regression analysis was performed using the lme4 package in R (R Foundation for Statistical Computing). All other statistical tests were performed by GraphPad Prism, version 8.2.1 (GraphPad Software).

## 3 RESULTS

### 3.1 Plasma CBS activity assay validation

**FIGURE 1:**
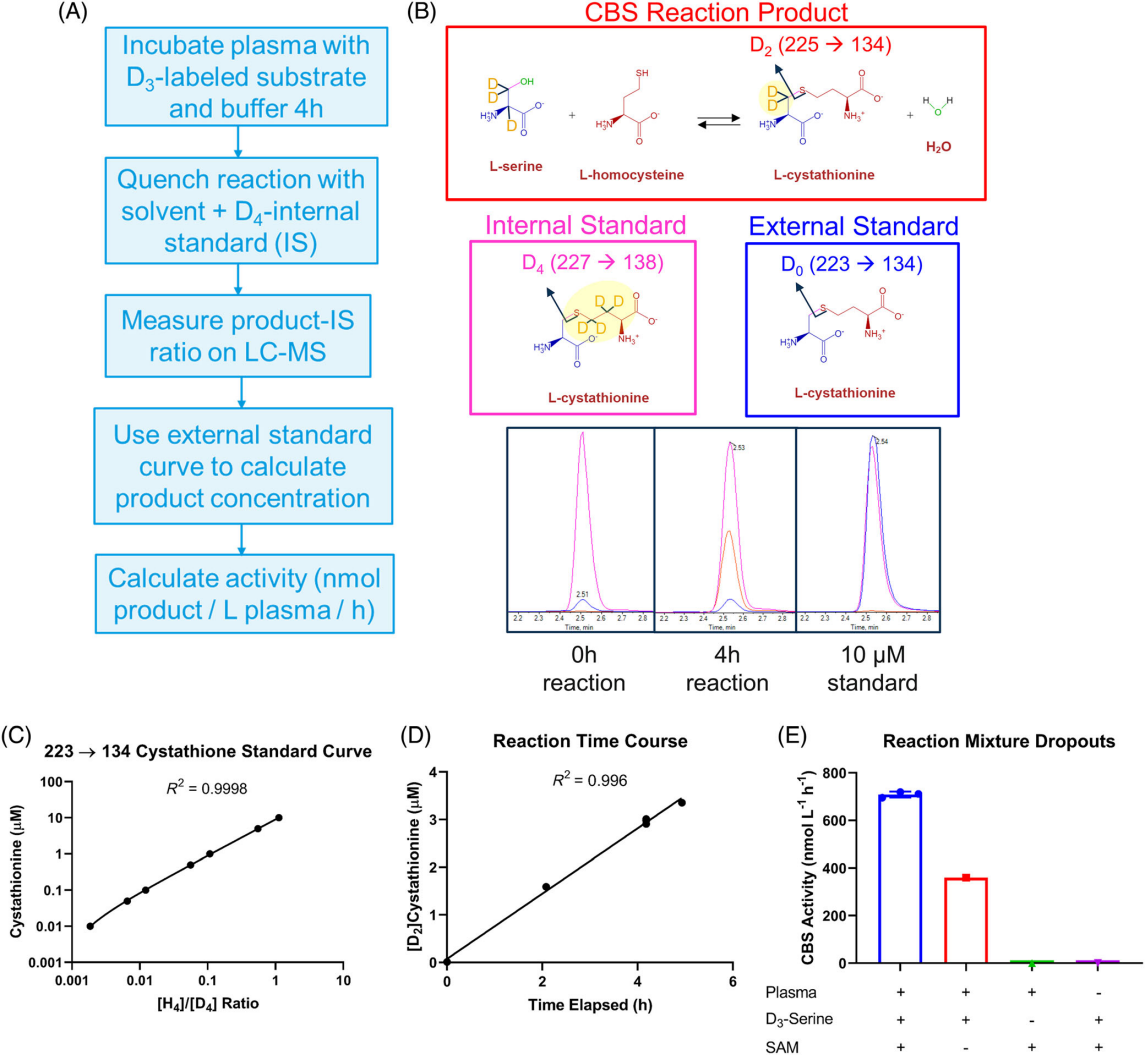
Plasma cystathionine β‐synthase (CBS) activity assay overview and validation controls. (A) Assay workflow overview; (B) overview of cystathionine species quantified on liquid chromatography‐mass spectrometry (LC–MS/MS), along with representative multiple reaction monitoring extracted ion chromatograms for different samples; D_2_‐cystathionine is formed as a reaction product from D_3_‐serine and homocysteine, D_4_‐cystathionine was used as an internal standard, and monoisotopic (D_0_ or H_4_) cystathionine was used as an external standard; (C) standard curve of the absolute concentration of cystathionine plotted against the peak area ratio of monoisotopic cystathionine to that of D_4_‐cystathionine; (D) D_2_‐cystathionine plotted against elapsed time; (E) CBS activity in reaction mixtures when either plasma, D_3_‐serine, or S‐adenosylmethionine (SAM) were excluded. R^2^ values correspond to unweighted and 1/Y‐weighted linear regression in (C) and (D), respectively.

Our LC–MS/MS‐based CBS measurement workflow is based on the protocol described by Krijt et al.^24^ (Figure 1A). The use of a stable isotope‐labeled substrate (D_3_‐serine) allows distinction of the CBS reaction product (D_2_‐cystathionine) produced over the course of the reaction from endogenous unlabeled cystathionine present in plasma. The calibration curve is based on the ratio of unlabeled external standards to stable isotope‐labeled internal standard (D_4_‐cystathionine; Figure 1B). These standard curves are linear and nominal standard concentrations fall within 15% of concentrations predicted by D_0_‐ (i.e., H_4_‐ or monoisotopic) cystathionine/D_4_‐cystathionine ratio (Figure 1C). As well, the concentration of D_2_‐cystathionine produced is linear over duration of the reaction (Figure 1D). As expected, no CBS activity was measured when plasma or D_3_‐serine substrate was removed from the reaction mixture (Figure 1E). Samples in which SAM was removed demonstrated that SAM enhanced measured CBS activity 2.0‐fold, similar to the 2.2‐fold increase previously reported.^24^ While no reference human plasma samples were used to link our CBS activity measurements to those published, the 709 nmol L^−1^ h^−1^ measured in commercially available human plasma fell within the range of 66–1066 nmol L^−1^ h^−1^ (mean: 404 nmol L^−1^ h^−1^, median 434 nmol L^−1^ h^−1^) reported in control samples by Krijt, et al.^24^

### 3.2 Plasma CBS activity is decreased in givosiran‐treated patients

**FIGURE 2:**
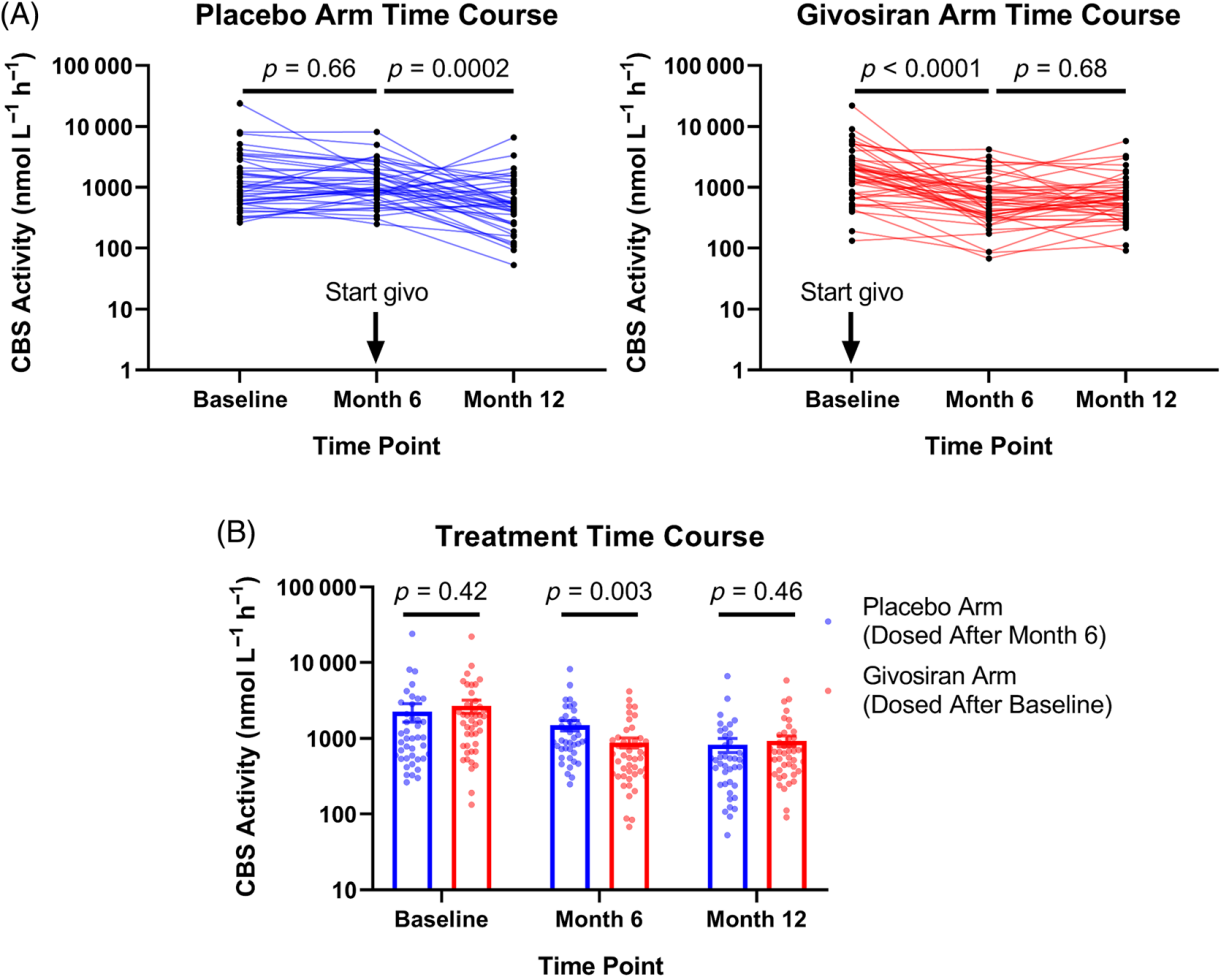
Plasma cystathionine β‐synthase (CBS) activity in placebo and givosiran arms across ENVISION study. (A) Individual patient trajectories of CBS activity measurements in placebo (left) and givosiran (right) treatment arms over time; (B) bar plots of CBS activity from each arm over time, with bar heights and error bars representing mean ± SEM. The *p*‐values result from Sidak's multiple comparisons test on log‐transformed values following two‐way analysis of variance.

At 6 months, a statistically significant decrease in median plasma CBS activity from baseline was observed in patients treated with givosiran (1734 nmol L^−1^ h^−1^ [baseline] vs. 557 nmol L^−1^ h^−1^ [at 6 months], *p* < 0.0001) but not in patients treated with placebo (1003 nmol L^−1^ h^−1^ [baseline] vs. 939 nmol L^−1^ h^−1^ [at 6 months], *p* = 0.66; Figure 2 and Table S1). Following this 6‐month double‐blind period, an open‐label extension period began, during which patients in the placebo arm were given givosiran. Patients in the placebo arm had a statistically significant decrease in median plasma CBS activity at 12 months (521 nmol L^−1^ h^−1^, *p* = 0.0002) into the trial, corresponding to 6 months after starting givosiran treatment. There was no evidence of a further decrease of CBS activity beyond 6 months of givosiran treatment based on comparison of (1) median CBS activity in the givosiran arm at Months 6 and 12 (557 and 666 nmol L^−1^ h^−1^, respectively, *p* = 0.68; Figure 2A) (2) median CBS activity in the givosiran arm at 12 months and the placebo arm at 6 months of treatment (666 and 521 nmol L^−1^ h^−1^, respectively; Figure 2B).

### 3.3 Plasma CBS activity correlates with homocysteine levels in givosiran‐treated patients

**FIGURE 3:**
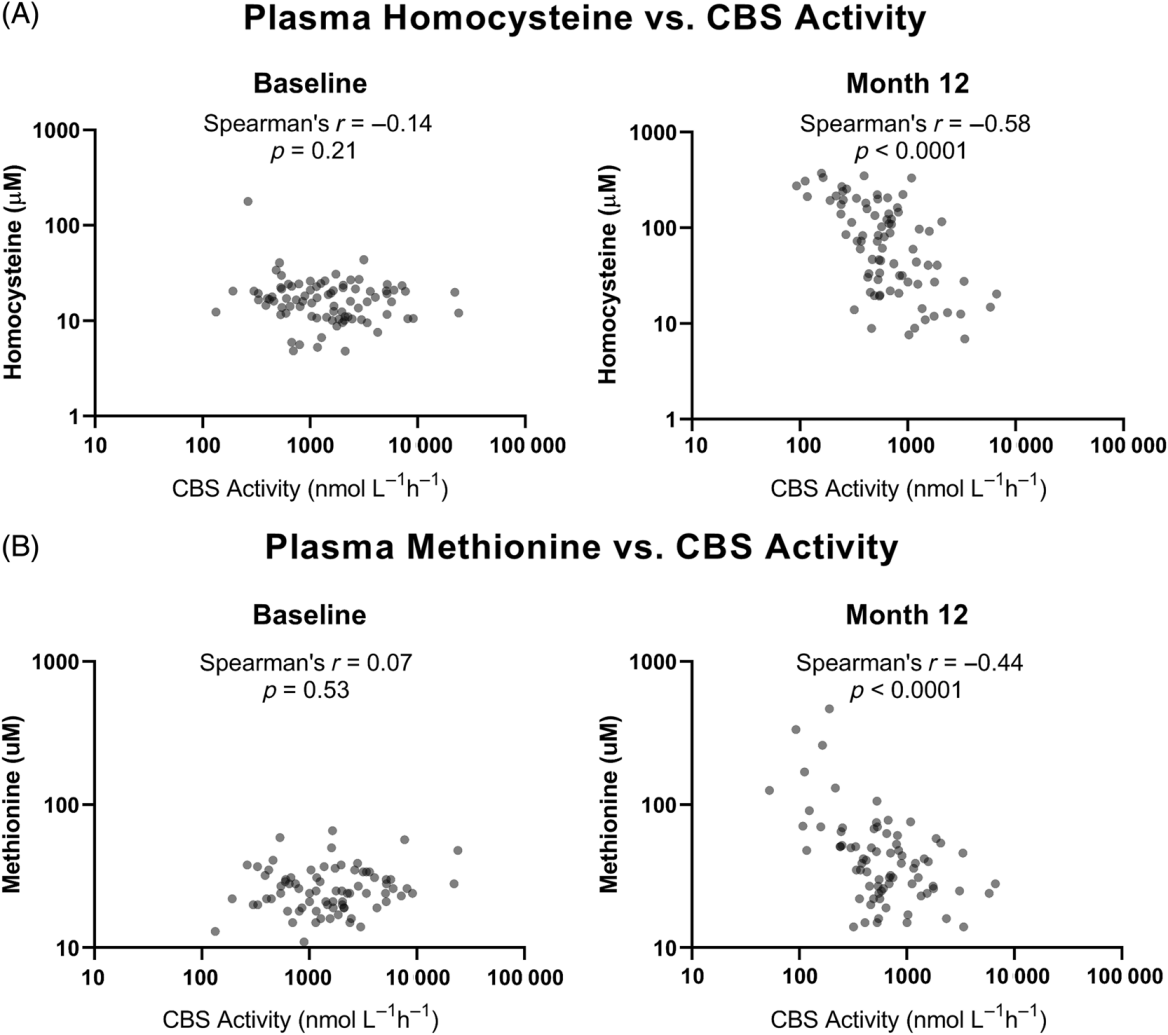
Correlation between plasma cystathionine β‐synthase (CBS) activity and plasma (A) total homocysteine and (B) methionine concentrations. Baseline and Month 12 values are given by the left and right panels, respectively. The *p*‐values correspond to two‐tailed test for nonparametric (Spearman's) correlation.

At the baseline timepoint, there was a relatively low statistically significant correlation between homocysteine levels and CBS activity, depending on whether all patients were considered together or grouped by trial arm (Figures 3A and S1a and Tables S1 and S2). However, following givosiran treatment, this correlation was found to be stronger and statistically significant (or more significant, in the case of the placebo arm). These changes in correlation and statistical significance were observable when comparing both Month 12 versus baseline among all patients (Figure 3A) and also givosiran‐treated versus placebo‐treated patients at Month 6 (Figure S1a). (The measured homocysteine concentration exceeded the quantification limit of the assay for five samples, and the CBS activities of these samples were among the lowest recorded, ranging from 68 to 108 nmol L^−1^ h^−1^.) There was no statistically significant correlation between methionine levels and CBS activity at baseline (Figures 3B and S1b and Table S3). However, as observed with homocysteine, the correlation between methionine levels and CBS activity was moderate and statistically significant in all patients who received givosiran at Month 12. As the givosiran arm (12 months of treatment) did not show higher correlation than placebo arm (6 months of treatment; Figure S1), there was no evidence of strengthening in the correlation between CBS activity and homocysteine or methionine levels with longer givosiran treatment.

Individuals with elevated alanine transaminase (ALT)/aspartate transaminase (AST) were previously described to also have elevated CBS activity levels, likely because it is also an intracellular enzyme released due to leakage from the liver.^24^ Consistent with this previous report, we observed significant positive associations between ALT and AST levels and CBS activity at baseline (Figure S2). While these correlations persisted following givosiran treatment, they weakened considerably, suggesting the emergence of other factors such as givosiran treatment that contributed to the observed variance. To verify that homocysteine levels still correlated significantly with CBS activity levels, even when accounting for ALT/AST levels, linear regression analysis was used either with ALT or AST or without. Statistically significant correlations were observed between homocysteine levels and CBS Activity at Month 12 both without (*p*‐value = 0.00012) or with ALT or AST (*p*‐values = 0.00048 and 0.00028, respectively).

### 3.4 Plasma CBS activity is normalized postsupplementation with vitamin B_6_

Four patients with elevated homocysteine levels started a daily multivitamin supplement containing 3 mg vitamin B_6_ as pyridoxine during the course of the ENVISION study. These four patients received vitamin B_6_ supplementation anywhere from 32 to 34 months into the study and subsequently experienced reductions in their plasma homocysteine levels (Table S4). The closest timepoints before and after vitamin B_6_ supplementation were collected at Months 24 and 36; therefore, these samples were analyzed for CBS activity.

**FIGURE 4:**
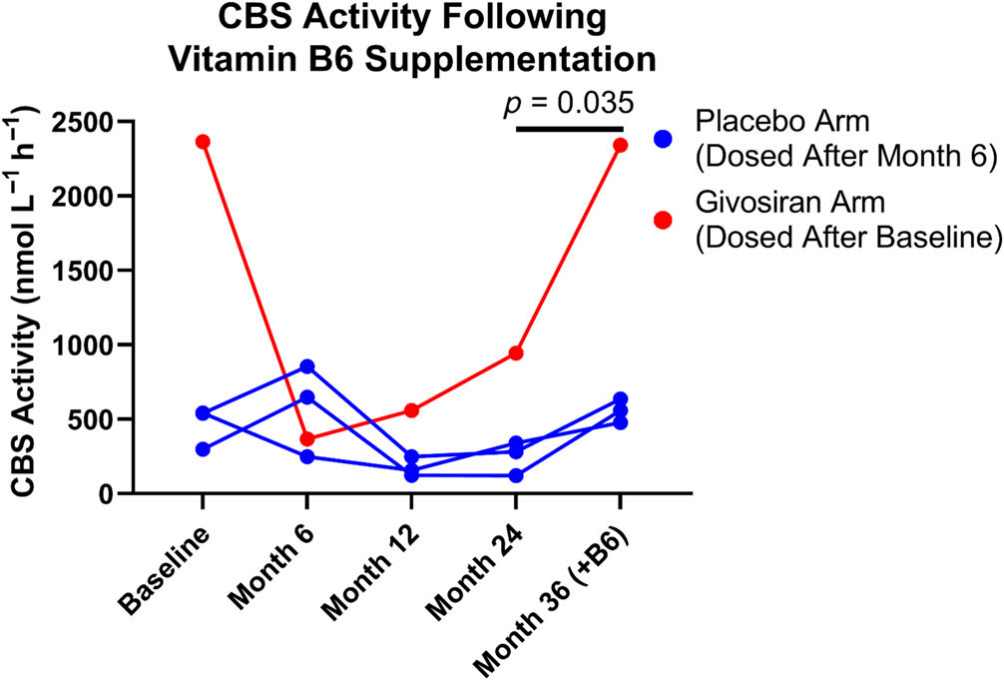
Plasma cystathionine β‐synthase (CBS) activity values in four patients over time prior to and following supplementation with vitamin B_6_. The month 24 versus month 36 *p*‐value results from two‐tailed paired *t*‐test on log‐transformed value.

CBS activity trended upward following vitamin B_6_ supplementation (Figure 4). All patients' measured CBS activities increased following supplementation, and while the sample size is small, this increase in CBS activity from Months 24 to 36 is statistically significant.

## 4 DISCUSSION

As demonstrated by Krijt et al.^24^ to distinguish healthy individuals from patients with loss‐of‐function CBS mutations, the plasma CBS assay reported here reflects underlying CBS activity, assumed to derive predominantly from the liver. The assay uses a stable isotope‐labeled substrate which produces a labeled reaction product that can be readily differentiated via LC–MS/MS from endogenous cystathionine (Figure 1B). Validation samples generated results similar to those observed in the original published protocol.^24^ For instance, reaction product formation was constant and linear over the course of the assay (Figure 1D), and product formation was dependent on the CBS cosubstrate serine, the plasma enzyme source, and the allosteric activator SAM (Figure 1E).

Elevated homocysteine levels observed with givosiran treatment were hypothesized to be due to lowering of CBS activity due to the corresponding elevations in methionine levels. Direct measurement of circulating CBS activity in patients from the Phase III ENVISION study confirms that CBS activity is reduced post‐givosiran treatment (Figure 2). However, in contrast to patients with classical homocysteinemia, plasma CBS activity never reached undetectable levels following givosiran treatment.^24^ While we cannot discount the potential influence of interlaboratory differences (e.g., sample handling/storage, instrument sensitivity), this observation suggests a reduction rather than complete abolishment of CBS activity with givosiran treatment. Plasma CBS activity was found to inversely correlate with homocysteine and methionine levels in givosiran‐treated patients (Figures 3 and S1). Consistent with the role of homocysteine as a substrate for CBS, these changes suggest that givosiran‐induced homocysteine elevation is due to a decrease in CBS activity. While there was a positive correlation of ALT and AST levels with plasma CBS, as previously observed,^24^ the negative correlation between CBS activity and homocysteine following givosiran treatment was still significant when accounting for levels of either transferase via multivariable linear regression. In fact, the greater magnitude correlation coefficient observed with homocysteine at the 12‐month time point suggests a stronger association between CBS and homocysteine compared with ALT/AST.

Plasma CBS activity levels were restored to pre‐givosiran treatment levels upon supplementation with a multivitamin preparation containing vitamin B_6_, a cofactor for CBS,^19^ in the four patients where such samples were collected. Our results are consistent with the reduction in homocysteine levels in AHP patients treated with givosiran following vitamin B_6_ supplementation described in Ventura et al.^11^ Expert opinion recommends monitoring total plasma homocysteine and vitamin B_6_, B_12_, and folate levels before and during givosiran treatment; supplementing with vitamin B_6_ in patients with homocysteine levels above 100 μM; and discussing the option of supplementing with vitamin B_6_ with patients with homocysteine levels above 30 μM.

What leads to lower CBS activity remains unknown. Givosiran lowers ALAS1 messenger RNA, potentially causing a lowering of bioavailable heme in the liver. Human CBS requires two cofactors for function, PLP and heme. The CBS enzyme contains a heme‐binding motif that has been suggested to stabilize the enzyme.^19^, ^26^ It is likely that supplementation with vitamin B_6_ compensates for lower bioavailable heme pool, as following supplementation, CBS activity levels return to baseline in the four patients where data is available (Figure 4), an effect similar to that observed in individuals with certain CBS mutations. However, other factors may affect baseline levels of homocysteine or CBS activity, as evidenced by elevated homocysteine in a subset of AHP patients even in the absence of givosiran treatment.

## 5 CONCLUSION

Using an LC–MS/MS‐based assay,^24^ we found plasma CBS activity in AHP patients to be reduced following givosiran treatment in samples collected from the Phase III ENVISION clinical trial.^6^ Plasma homocysteine and methionine, previously reported to shift upward in givosiran‐treated patients,^11^ were found to be inversely correlated with CBS activity. In four patients given supplements containing vitamin B_6_, CBS activity trended upward within 2–4 months following supplement initiation. Overall, these results support the hypothesis that elevated homocysteine levels following givosiran treatment result from reduction of CBS activity and can be mitigated by vitamin B_6_ supplementation.

## AUTHOR CONTRIBUTIONS

Mark A. Keibler performed experiments. Mark A. Keibler and Simina Ticau developed the study methodology, performed data analysis and visualization, and drafted the original article. Gautham V. Sridharan, Marianne T. Sweetser, and Simina Ticau supervised the study. All authors contributed to the conception and design of the study, interpreted the data, and revised and approved the article.

## FUNDING INFORMATION

This study was sponsored by Alnylam Pharmaceuticals.

## CONFLICT OF INTEREST STATEMENT

All authors are employees and stockholders of Alnylam Pharmaceuticals.

## ETHICS STATEMENT

The ENVISION study (NCT03338816) followed the guidelines of the International Conference on Harmonization, the World Health Organization Declaration of Helsinki, and the Health Insurance Portability and Accountability Act of 1996. The study protocol and amendments were approved by institutional review boards and ethics committees at each study site.

## INFORMED CONSENT STATEMENT

Written informed consent was obtained from all patients.

## PATIENT CONSENT STATEMENT

Informed consent was obtained from all subjects involved in the study.

## ANIMAL RIGHTS

This article does not contain any studies with animal subjects performed by any of the authors.

## Supporting information


**Data S1.** Supporting information.

## Data Availability

Individual participant data that support the ENVISION study results are available for data requests in a secure‐access environment. Access will be provided contingent upon the approval of a research proposal and the execution of a data sharing agreement. Requests for access to data can be submitted via the website www.vivli.org.
