# Supplementary material for: Elevated homocysteine is negatively correlated with plasma cystathionine β‐synthase activity in givosiran‐treated patients
Source: JIMD Rep. 2024 Apr 29;65(4):262–71. doi: 10.1002/jmd2.12416 (PMC11224493; doi:10.1002/jmd2.12416)
Supplement: Supplementary file 1 — Data S1. Supporting information. [file JMD2-65-262-s001.docx]

*
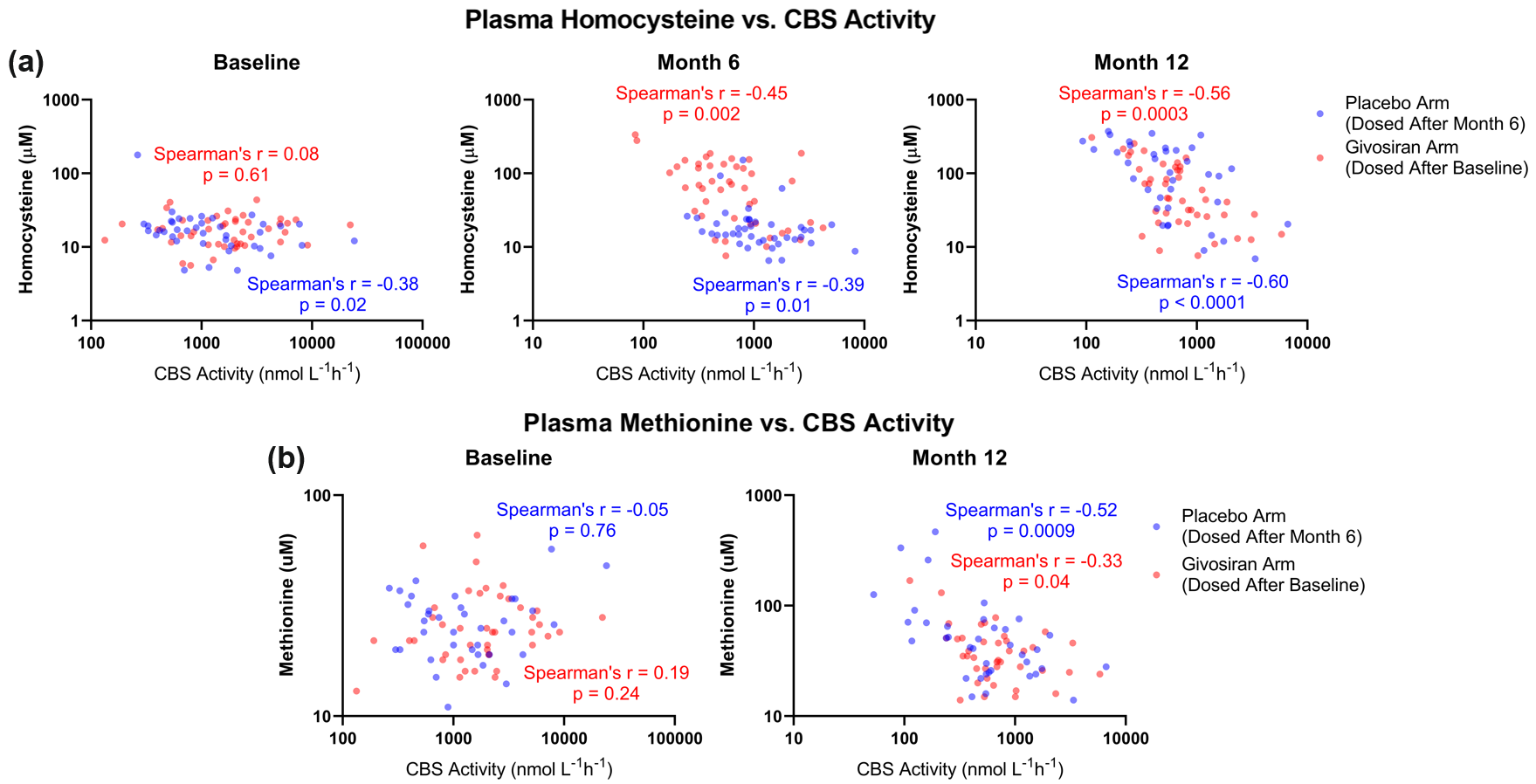
*

**Supplementary Figure 1.** Correlation between plasma CBS activity and plasma (a) total homocysteine (left, center, and right panels show baseline, month 6, and month 12 values, respectively) and (b) methionine concentrations (left and right panels show baseline and month 12 values, respectively). Data from placebo and givosiran arms are labeled separately. P-values correspond to two-tailed test for non-parametric (Spearman’s) correlation.

**
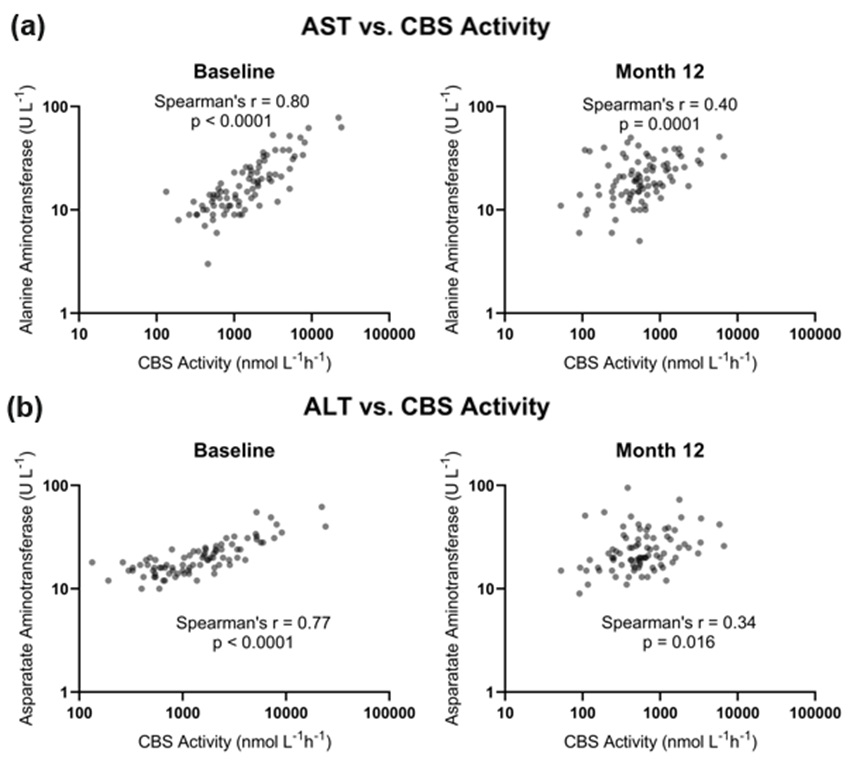
**

**Supplementary Figure 2.** Correlation between plasma CBS activity and plasma (a) alanine amino transferase (ALT) and (b) aspartate aminotransferase (AST) levels. Baseline and month 12 values are given by the left and right panels, respectively. P-values correspond to two-tailed test for nonparametric (Spearman’s) correlation.

**Supplementary Table 1.** Summary statistics of CBS activity across treatment arms and time points.

| **Treatment Arm** | **Time Point** | **N** | **CBS activity (nmol L^-1^ h^-1^)** | | |
| --- | --- | --- | --- | --- | --- |
|  |  |  | **Median** | **Interquartile range** | **Range** |
| **Placebo** | Baseline | 41 | 1003 | 545-2843 | 264-24106 |
|  | Month 6 | 41 | 939 | 687-1791 | 249-8227 |
|  | Month 12 | 41 | 521 | 247-993 | 53-6644 |
| **Givosiran** | Baseline | 45 | 1734 | 800-2726 | 133-22205 |
|  | Month 6 | 45 | 557 | 328-980 | 68-4227 |
|  | Month 12 | 45 | 666 | 375-1013 | 91-5842 |

**Supplementary Table 2.** Summary statistics of homocysteine levels across treatment arms and time points.

| **Treatment Arm** | **Placebo** | | | **Givosiran** | | |
| --- | --- | --- | --- | --- | --- | --- |
| **Time Point** | **Baseline** | **Month 6** | **Month 12** | **Baseline** | **Month 6** | **Month 12** |
| **N** | 40 | 41 | 40 | 45 | 45 | 45 |
| **Median (μM)** | 16.6 | 16.2 | 127.4 | 17.3 | 69.3 | 46.3 |
| **Interquartile range (μM)** | 11.4-21.0 | 12.5-22.8 | 35.7-236.6 | 11.1-22.7 | 21.3-130.3 | 26.6-122.5 |
| **Range (μM)** | 4.8-178.3 | 6.5-151.6 | 6.9->400.0 | 5.6-47.8 | 7.6->400 | 7.7->400 |

**Supplementary Table 3.** Summary statistics of methionine levels across treatment arms and time points.

| **Treatment Arm** | **Placebo** | | **Givosiran** | |  |
| --- | --- | --- | --- | --- | --- |
| **Time Point** | **Baseline** | **Month 12** | **Baseline** | **Month 12** | |
| **N** | 36 | 37 | 42 | 40 | |
| **Median (μM)** | 26.5 | 44 | 24.0 | 35.0 | |
| **Interquartile range (μM)** | 20.0-33.5 | 25.5-70.5 | 19.0-31.0 | 25.3-50.8 | |
| **Range (μM)** | 11.0-57.0 | 14.0-466.0 | 13.0-66.0 | 14.0-169.0 | |

**Supplementary Table 4.** Plasma homocysteine concentrations in four patients over time prior to and following administration of a supplement containing vitamin B6 (see Ventura *et al*., 2022).

| **Patient/Treatment Arm*** | **Visit** | **Homocysteine (μM)** |
| --- | --- | --- |
| Patient 1  Placebo | Baseline | 30.2 |
|  | Month 6 | 23.9 |
|  | Month 12 | 241.0 |
|  | Month 24 | 320.9 |
|  | Month 36 | 30.0 |
| Patient 2  Placebo | Baseline | 21.7 |
|  | Month 6 | 26.2 |
|  | Month 12 | 372.2 |
|  | Month 24 | 356.4 |
|  | Month 36 | 96.4 |
| Patient 3  Placebo | Baseline | 20.6 |
|  | Month 6 | 15.1 |
|  | Month 12 | > 400.0 |
|  | Month 24 | 331.9 |
|  | Month 36 | 46.9 |
| Patient 4  Givosiran | Baseline | 16.0 |
|  | Month 6 | 41.7 |
|  | Month 12 | 45.5 |
|  | Month 24 | 49.9 |
|  | Month 36 | 18.8 |

* Patients in the placebo arm began givosiran treatment at Month 6. Both the baseline and Month 6 homocysteine measurements were taken from plasma samples collected prior to dosing.

Ventura P, Sardh E, Longo N, et al. Hyperhomocysteinemia in acute hepatic porphyria (AHP) and implications for treatment with givosiran. *Expert Rev Gastroent*. 2022;16(9):879-894. doi:10.1080/17474124.2022.2110469
